# Photonic Crystal Based Sensor for Organic Solvents and for Solvent-Water Mixtures

**DOI:** 10.3390/s121216954

**Published:** 2012-12-12

**Authors:** Christoph Fenzl, Thomas Hirsch, Otto S. Wolfbeis

**Affiliations:** Institute of Analytical Chemistry, Chemo- and Biosensors, University of Regensburg, 93040 Regensburg, Germany; E-Mails: christoph.fenzl@ur.de (C.F.); thomas.hirsch@ur.de (T.H.)

**Keywords:** photonic crystal, sensor, PDMS, solvent polarity, water sensor

## Abstract

Monodisperse polystyrene nanoparticles with a diameter of 173 nm were incorporated into a polydimethylsiloxane matrix where they display an iridescent color that can be attributed to the photonic crystal effect. The film is of violet color if placed in plain water, but turns to red in the presence of the non-polar solvent *n*-hexane. Several solvents were studied in some detail. We show that such films are capable of monitoring the water content of ethanol/water mixtures, where only 1% (v/v) of water leads to a shift of the peak wavelength of reflected light by 5 nm. The method also can be applied to determine, both visually and instrumentally, the fraction of methanol in ethanol/methanol mixtures. Here, a fraction of 1% of methanol (v/v) results in a wavelength shift of 2 nm. The reflected wavelength is not influenced by temperature changes nor impeded by photobleaching. The signal changes are fully reversible and response times are <1 s.

## 1. Introduction

The determination of water in organic solvents is of highest significance in industry, for example the pharmaceutical and chemical, as the presence of water often impedes not only the production of chemical and drugs, but also the efficiency of drugs and the usefulness of chemical products. The Karl Fischer titration [1] is the most widely used method for the determination of water in solvents, liquid foods, and the like. This method, while being the gold standard, has disadvantages such as need of expensive chemicals and electrodes, the need for specialized instruments and water-free titration cells, and the fact that it can only be performed by trained personnel. In addition, it is time consuming and easily disturbed by interfering compounds. Other methods include chromatography [2] and spectroscopic techniques [3,4] based on fluorescent probes [5–9]. The latter sometimes suffer from photobleaching of the probes. Langhals [10] has introduced a very sensitive photometric procedure for the determination of water in organic solvent using the strongly solvatochromic probe pyridinium phenolate betaine. However, this method—while sensitive—does not enable continuous sensing.

Alcoholic beverages must not contain methanol. Unfortunately, spirits produced in home distilleries can be contaminated with methanol which is the byproduct of the mashing process. The lethal dose by ingestion is often assumed to be 100 mL of pure methanol, but cases have also been reported where only 15 mL of 40% (v/v) methanol have caused fatalities. Quantities as small as 4 mL of pure methanol can lead to blindness [11]. Recently, a Bragg grating sensor has been presented that enables mixtures of organic (mainly alcoholic) liquids to be analyzed [12]. The method is fast but the overall signal change is small, typically 1 nm only over the whole concentration range of ethanol/methanol mixtures. Quicker and more simple methods are sought, ideally such that the concentration can be visually estimated.

We present here a solvent-responsive sensor film based on the use of photonic crystal (PhC) technology [13]. PhCs consist of periodically arranged and regularly shaped, mostly transparent, materials consisting of nanoparticles and a matrix polymer, both of which differ in their dielectric constants. In perfect PhCs, light of only a certain wavelength can propagate through the crystal lattice [14–17]. Three-dimensional PhCs are characterized by a periodicity that extends into three dimensions. Major advances in the field of sensors based on PhCs were achieved by the group of Asher [18,19]. The sensor film presented here consists of drop-cast polystyrene nanoparticles incorporated into a polydimethylsiloxane (PDMS) matrix [20,21]. Notwithstanding recent developments in sensors based on elastomeric photonic crystals [22–24] and in sensing alcohols via PhCs [25–28], the colloidal crystal array (PCCA) films presented here excel due to their simple synthesis and handling, and by their very distinct sensitivity to solvent polarity as will be shown below. They enable the fraction of water in ethanol/water mixtures, or the fraction of methanol in ethanol/methanol mixtures to be determined. This approach possesses advantages such as fast response (<1 s), full reversibility of the signal change, and complete lack of photobleaching.

## 2. Experimental Section

### 2.1. Preparation of a Polydimethylsiloxane Matrix-Based Colloidal Photonic Crystal Array Film

**Figure 1.**
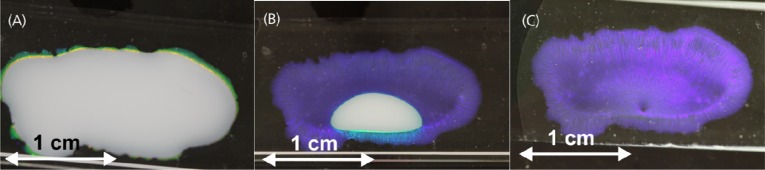
Photo of drop-cast polystyrene microspheres, and effect of evaporation of water. (**A**) after 24 h; (**B**) after 48 h; (**C**) after 72 h.

Microscope glass slides were first treated with a concentrated solution of potassium hydroxide (Merck, Darmstadt, Germany) in ethanol and then used as substrates. After thorough washing, 500 μL of a dispersion of commercial monodisperse polystyrene microspheres (173 ± 6) nm in diameter, a 2.5% w/v suspension (Microparticles GmbH, Berlin, Germany) were placed in the center of the glass substrate. The drop was encircled in a ring of PDMS oil with a viscosity of 10 cSt (from ABCR GmbH, Karlsruhe, Germany) to allow slow evaporation. This silicone oil was also placed on top of the aqueous drop for the same purpose. After three days, all the water was evaporated and a purple colored layer of photonic crystals can be seen on the substrate (Figure 1).

**Figure 2.**
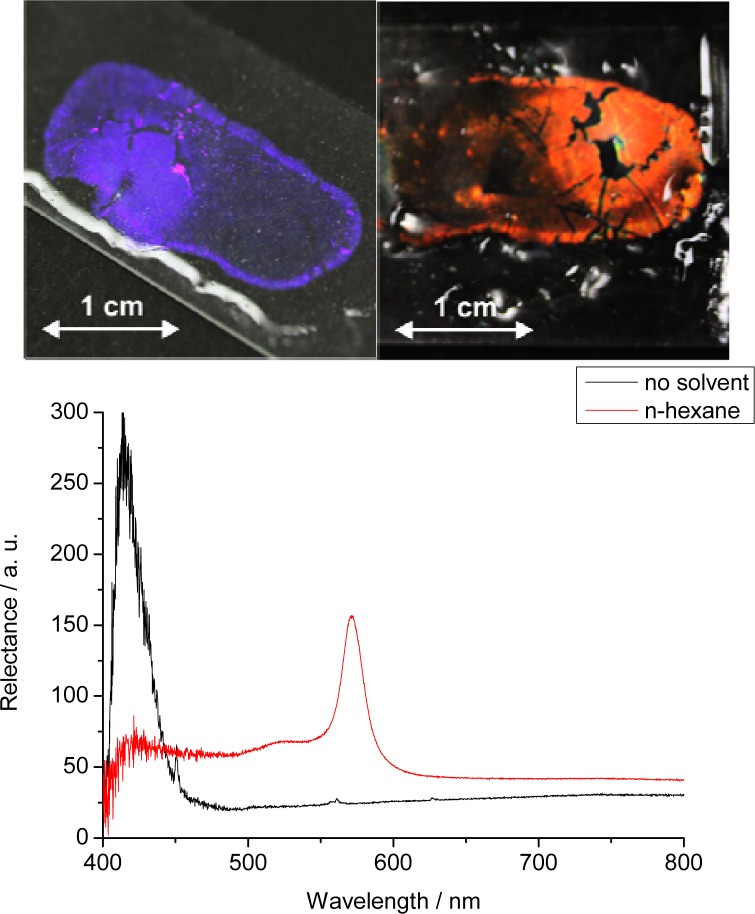
Photographs and reflection spectra of polystyrene nanoparticles incorporated into a polydimethylsiloxane matrix. Left (violet): before addition of *n*-hexane. Right (red): after addition. The black areas in the films are due to damage when manually peeling off excess of PDMS on the photonic crystals.

The silicone oil was carefully dabbed away with a paper towel. The PhC array was then stabilized by a polydimethylsiloxane polymer. The polymerization mixture consisted of Sylgard 184 base component (3 g, Dow Corning, Midland, MI, USA), *n*-hexane (3 g, Acros, Geel, Belgium), and Sylgard 184 curing agent (0.65 g, Dow Corning). After thorough stirring, the mixture was poured over the photonic crystals. After one day of curing in air, the glass slide was heated to 60 °C in an oven for 1 h to complete polymerization. Excess of PDMS over the photonic crystals was cautiously peeled off using tweezers. This process can easily lead to damage if areas are larger than 5 × 5 mm as can be seen in Figure 2. The resulting PCCA-film was tested for its response to various solvents [29].

### 2.2. Measurements of Reflected Light of Polymerized Colloidal Crystal Arrays

A volume of 500 μL of an organic solvent such as methanol, ethanol, PDMS oil (with a viscosity of 0.65 cSt.; from ABCR) and *n*-hexane (Acros), and various mixtures of solvents were placed on the photonic crystal film. The effect of temperature was studied between 4 °C and 100 °C. A xenon lamp (0.5 W) was for illumination, with an optical fiber waveguide fixed at an angle of 90° with respect to the lamp. The fiber was connected to an USB 4000 spectrometer (Ocean Optics, Dunedin, FL, USA). Reflection spectra were recorded and analyzed with the SpectraSuite software (Ocean Optics) in the reflectance mode and an integration time of 100 ms per spectrum.

## 3. Results and Discussion

Polydimethylsiloxane (PDMS) is an elastic polymer that swells or shrinks depending on the polarity of the solvent it is in contact with. Swelling can be expressed by a swelling parameter *S* defined as:

$$S=D/D_{0}$$

where *D* is the length of a PDMS film in the solvent and *D_0_* is the length of the dry PDMS [30]. Any change in length and—simultaneously—of volume will alter the distance between the nanoparticles with respect to one another. This causes a change of the wavelength of the reflected light.

### 3.1. Sensitivity to Organic Solvents

First tests were performed with the highly nonpolar organic solvent *n*-hexane. The color change can be clearly identified with the bare eye as can be seen in Figure 2. We believe that this sensor film can be used to detect various kinds of nonpolar solvents but of course is not applicable to solvents that can dissolve polystyrene, examples being acetone. If such solvents are to be detected, we suggest to exchange the polystyrene nanoparticles by a more inert material such as silica spheres. Table 1 specifies the solvents tested, and their effects on the maximum of the reflected wavelength.

**Figure 3.**
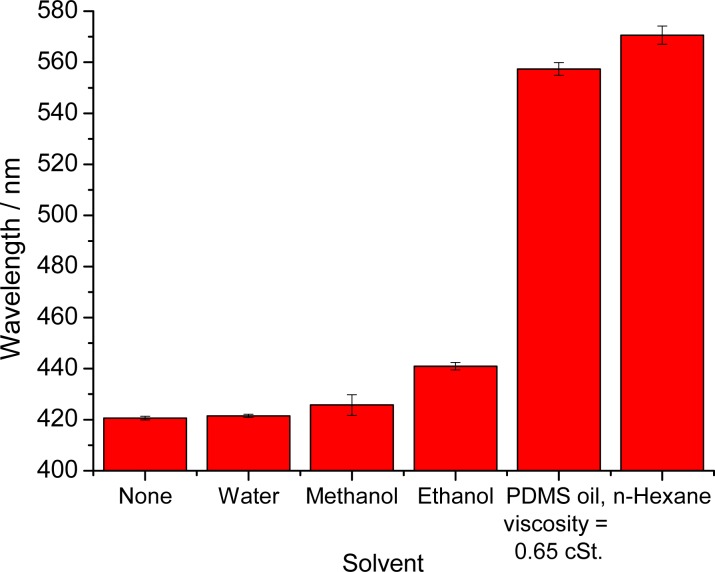
Effect of various solvents on the wavelength of reflected light of a polymerized colloidal crystal array consisting of polystyrene nanoparticles in PDMS. The data are average values obtained in experiments with five different sample films.

Figure 3 gives a plot of the maximum of the reflected wavelength versus the relative permittivity of the respective solvent. One can see that the complete range from high polarity (water) to very low polarity (*n*-hexane) is covered by the visible range of the wavelength of the reflected light of the PhC.

It is known that a decrease in the polarity of the solvent goes in parallel with a decrease of the relative permittivity and—thus—an increase of the swelling parameter. This relation was corroborated here in that the wavelength of maximally reflected light shifts to the red as the volume of the film increases. The largest shifts are observed with the nonpolar solvent *n*-hexane. However, even in the presence of ethanol, which is relatively polar, the signal change can be visually detected, whereas water and methanol do not cause a substantial effect. The results illustrate the potential of a hydrophobic PCCA-film for monitoring the composition of solvent mixtures.

### 3.2. Ethanol/Water Mixtures

**Figure 4.**
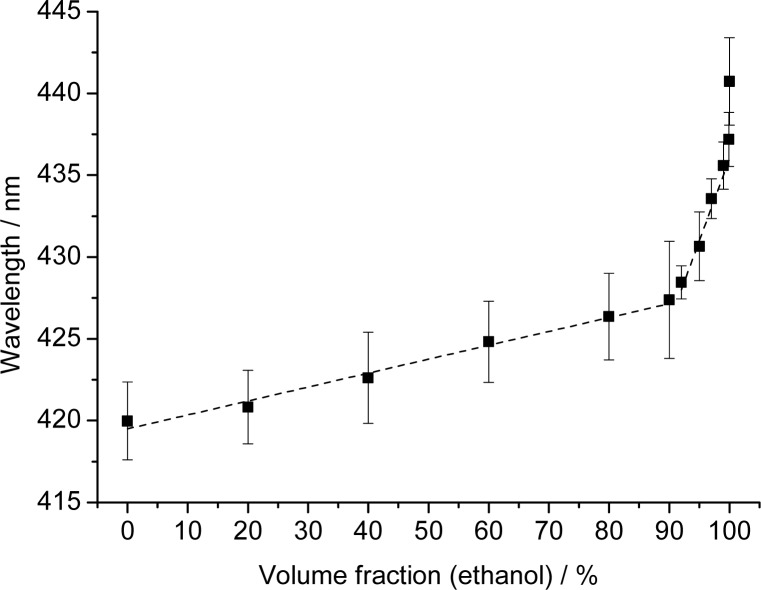
Effect of the fraction of ethanol in water on the peak wavelength of light reflected by PCCA films consisting of polystyrene nanoparticles in polydimethylsiloxane. The error bars show the standard deviation of the evaluation from five individual experiments with the same PCCA film.

Given the strong response of the PCCA-film to changes in polarity, we have evaluated mixtures of water and ethanol. Reflection spectra were recorded for water, various water/ethanol mixtures, and for ethanol that was previously stored under ambient air and contained 0.03% (v/v) of water as verified by Karl Fischer titration. The results of the reflectometric measurements are shown in Figure 4.

The plot shows two linear regions. The first extends from 0 to 90% ethanol and is characterized by an only moderate increase in wavelength (∼7 nm). This part of the plot can be fit with the following equation: *y* = 0.085*x* + 419.5 (with a regression coefficient of 0.982). The second region (from 90 to 99% of ethanol) has a much steeper slope. This indicates better penetration of the membrane by ethanol which results in much stronger swelling of the polymer. This section can be fit by the equation *y* = 1.00*x* + 336 (with a regression coefficient of 0.986).

The results demonstrate that PhCs can be used for quick and simple sensing of the water fraction of ethanol because it works over the complete range of ratios. Polymerized hydroxyethyl methacrylate photonic crystals [27] showed similar effects but only up to fractions of 40% ethanol. Higher fraction could not be determined due to the limited stability of the methacrylate-based hydrogel. It has also been reported that inverse opal films are capable of detecting water-ethanol mixtures [25,28]. However, an arrangement of four differently functionalized inverse opal films is necessary in order to detect mixtures in the 85 to 100% ethanol range with a precision of 5% (which is moderate anyway). In yet another approach [26], an inverse opal polyacrylamide film was applied to sense the fraction of alcohols, but these PhCs also contain water and therefore cannot be used to detect small water fractions in almost pure alcohols. We believe that the sensing scheme presented here is not limited to the species and mixtures investigated here, but also envision that such (or similar) sensor films would enable the detection of traces of water in gasoline fuels.

### 3.3. Ethanol/Methanol Mixtures

**Figure 5.**
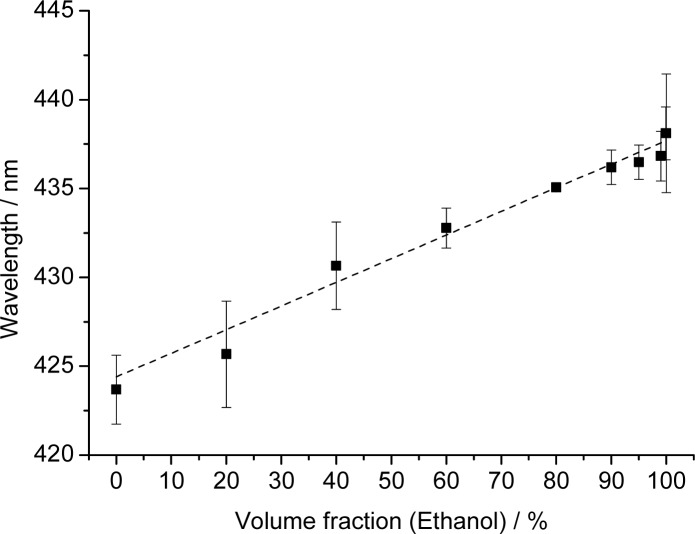
Effect of the fraction of ethanol in methanol on the peak wavelength of reflected light of polystyrene nanoparticles in polydimethylsiloxane. Each data point represents the average value of five measurements with the same PCCA film, and the error bars indicate the standard deviation.

As small amounts of water in ethanol can be easily detected by this PhC sensor, we also hoped to detect methanol in ethanol by this method. Respective experiments were performed, and the peak wavelengths of the reflected light are plotted versus the fraction of ethanol in methanol in Figure 5.

The data illustrate a linear dependence over the entire concentration range. This can be expected because methanol and ethanol are quite similar in terms of their chemical structure and in polarity. Both solvents can easily penetrate the PDMS matrix to cause swelling. This is not the case for water. The experimental data can be fit by the equation *y* = 0.133*x* + 424.4 (with a regression coefficient of 0.975). We note, however, a deviation from linearity between 99% and 100% ethanol which can be speculatively explained only. The system described by Pan *et al*. [26], (which is based on a polyacrylamide hydrogel inverse opal) also was used for the identification of various ethanol-methanol mixtures. However, it is limited by the fact that neither pure ethanol nor pure methanol can be detected. All mixtures have to contain at least 70% of water, and the maximum ratio of ethanol to methanol is 2:1.

### 3.4. Effect of Temperature

**Figure 6.**
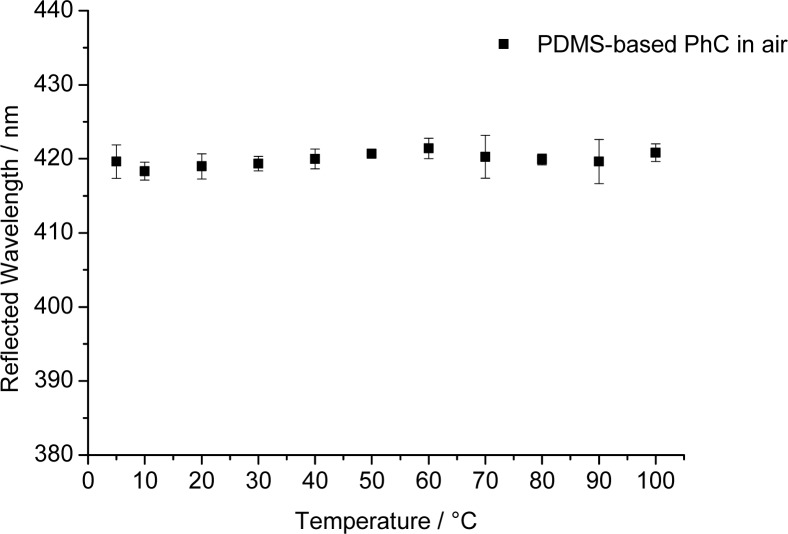
Effect of temperature on the peak wavelength of reflected light of the PDMS-based photonic crystal sensor, showing that it virtually has no effect. Data were acquired with a sensor film exposed to air.

Most chemical sensors are affected by temperature. We therefore have tested the PhC arrays with respect to the effect of temperature in the range from 5 °C to 100 °C and find—to our surprise—that the peak wavelength of the reflected light of 420 nm for air remains constant (±0.8 nm) over the whole temperature range (see Figure 6). This is a most welcome finding but difficult to interpret. Two opposite effects (thermal expansion and the effect of temperature on refractive index) may be operative: For the PDMS used here, the thermal expansion is approximately 3% in the temperature range under consideration [33]. The refractive index, in turn, becomes lower with increasing temperature. We assume that the two effects are compensating each other.

## 4. Conclusions and Outlook

We show here that PDMS-based photonic crystal arrays can be used to visually detect the presence of nonpolar solvents such as the hydrocarbon *n*-hexane, and probably numerous other hydrocarbons including gasolines. Depending on their polarity, solvents cause different degrees of swelling (and thus color shifts) of the sensor. If a sensor film equilibrated with humid air is exposed to methanol, a 5-nm shift is observed, while ethanol causes a 20-nm shift, which can already be visually detected. Less polar solvents such as PDMS oil and *n*-hexane lead to large longwave shifts (by 137 and 151 nm, respectively). We also show that by increasing the fraction of ethanol in ethanol/water mixtures results in a linear increase of the peak wavelength of reflected light. Similar studies were carried out with ethanol/methanol mixtures and revealed a virtually linear relationship between peak wavelength and the fraction of ethanol in methanol/ethanol mixtures. The signal changes reported here are completely reversible, and response times are as short as 1 s. We also note that such polymer films do not suffer from photobleaching as is the case of many fluorescence-based sensors. This clearly underpins the advantage of photonic crystal based sensing over indicator-based methods.

## Figures and Tables

**Table 1.** Reflected light of a thin film composed of polystyrene nanoparticles in polydimethylsiloxane in the presence of different solvents. The data for the relative permittivity of PDMS were taken from reference [31], those for the other solvents from reference [32]. The swelling parameters *S*, defined as the ratio of the polymer length in the solvent to the length of the dry polymer, are from reference [30]. The data given are average values obtained in experiments with five different sample films.

**Solvent**	**Relative permittivity ɛ_r_**	**Swelling parameter *S***	**Reflected wavelength/nm**	**Signal shift/nm**
None	-	-	420.6 ± 0.7	0
Water	78.36	1.00	421.4 ± 0.6	0.8
Methanol	32.66	1.02	426 ± 4	5
Ethanol	24.55	1.04	440.9 ± 1.4	20
PDMS oil ^a^	2.2	-	557 ± 2	137
*n*-Hexane	1.88	1.35	571 ± 3	151

a polydimethylsiloxane.
